# Serous leak, a rare complication of polytetrafluoroethylene grafts: a case report

**DOI:** 10.1186/1757-1626-2-195

**Published:** 2009-11-17

**Authors:** Gowreeson Thevendran, Rozanne Lord, Khaled M Sarraf

**Affiliations:** 1 Department of Surgery, The Hillingdon Hospital, Pield Heath Road, Uxbridge, Middlesex, London UB8 3NN, UK; 2 Department of Surgery, Royal Free Hospital, Pond Street, London NW3 2PN, UK

## Abstract

Perigraft seroma is a rare complication of polytetrafluoroethylene grafts especially in the lower limb. It is often difficult to treat and recurrence is common. We present a case of a young woman who sustained a persistent perigraft seroma following a polytetrafluoroethylene graft in the proximal thigh, which was implanted as an alternative to an arteriovenous fistula. The cause was not ascertained and this was eventually treated by complete removal of the graft. This case highlights an unusual complication of polytetrafluoroethylene grafts that are commonly used in vascular access surgery, which can be challenging to treat.

## Introduction

The use of polytetrafluoroethylene (PTFE) grafts has recently become an accepted alternative to an arteriovenous fistula in patients plagued with vascular access problems. Thrombosis and infection are amongst the major causes of morbidity reported in patients with PTFE grafts [1]. We report a rare case of a PTFE graft for vascular access complicated by a persistent serous leak predisposing to recurrent seromas and subcutaneous groin oedema [2,3].

## Case Presentation

We present a 31-year-old female British national of Asian ethnicity who was electively admitted for fashioning of a loop PTFE graft to the right thigh. Intra-operatively, the arterial and venous limbs of the graft were anastomosed end-to-side to the superficial femoral artery and femoral vein respectively. A good thrill and bruit were documented post-procedure.

Post operatively, swelling and tenderness was noted around the groin incision extending over the subcutaneous tunnel of the looped segment of the graft. In a fortnight, the thigh remained swollen and tender to touch. Intermittent temperature spikes were noted with a moderate leucocytosis and an elevated C-reactive protein (CRP). This was treated conservatively with broad spectrum intravenous antibiotics and compression stockings. Serial ultrasound scans confirmed a groin collection, subcutaneous oedema of the thigh and good flows in the graft. Two wound explorations were performed for evacuation of perigraft haematomas and washouts. Unfortunately, on each occasion, the swelling resumed.

As a means of symptomatic relief, a 14-gauge (14G) cannula connected to a free drainage system was inserted into the swelling. This drained 1.7 litres of serous fluid overnight. In suspect of a lymphatic leak, a pre-operative lymphangiogram was performed and the patient consented for surgery.

**Figure 1:**
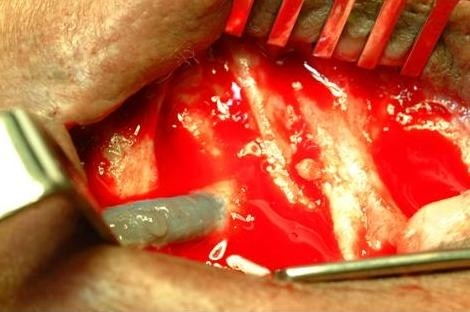
**Serous fluid accumulating on the graft surface**.

**Figure 2:**
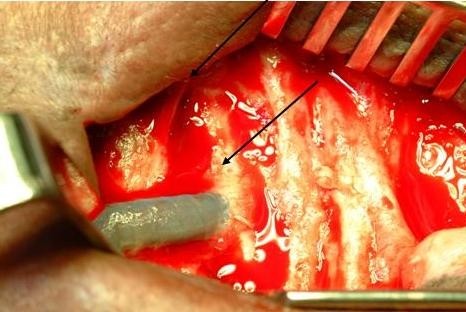
**Rapid re-accumulation of serous fluid on the graft surface**.

**Figure 3:**
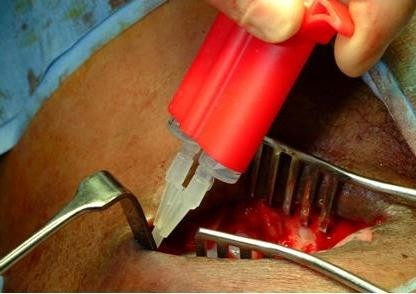
**Application of tissue glue to the condensing surface of the graft**.

Intra-operatively, we observed active oozing serum from a 3 cm portion of the arterial limb of the PTFE graft adjacent to the anastomotic site (Figure 1). This condensation of serous fluid was rapid and seems to occur only on the specified graft segment (Figure 2). A decision to apply tissue glue to this condensing surface of the graft was made in an attempt to cease further extravasation of serous fluid (Figure 3). Unfortunately, the swelling resumed 24 hours post-operatively and informed consent was obtained for graft removal. At operation, the glued patch was noted to have lifted off the surface of the graft. The limbs of the graft were disconnected and the entire loop segment removed.

## Discussion

Perigraft seroma is a rare complication of PTFE grafts. The aetiology of this enhanced graft permeability is unclear. It was suggested that the serous component of blood was seeping out of the graft lumen due to removal of the coiled rings on the graft surface. This was disputed by the graft manufacturers. Indeed, the site of serous leakage did not coincide with the position of the rings though it remains doubtful if similar condensations were occurring along the tunnelled segment of the graft. The use of intra-operative Betadine washouts was also questioned as a possible cause of increased graft permeability.

We have had two previous experiences with similar phenomena (unpublished). Of those, one was amenable to sealing with topical administration of haemostatic agents. The value of this case, in our opinion, is that it highlights an unusual complication of PTFE grafts that are commonly used in vascular access surgery.

## Competing interests

The authors declare that they have no competing interests.

## Authors' contributions

GT, RL and KMS have contributed to the conception, acquisition of data, analysis, and have been involved in drafting the manuscript and revising it; and have given final approval of the version to be published.

## Consent

Written informed consent was obtained from the patient for publication of this case report and accompanying images. A copy of the written consent is available for review by the Editor-in-Chief of this journal.
